# An Excellent Appointment

**Published:** 1898-07

**Authors:** 


### AN EXCELLENT APPOINTMENT.

GOVERNOR HASTINGS, of Pennsylvania, deserves much credit for his prompt recognition of the importance of a delegate to the Congress on Tuberculosis, at Paris, July 27th to August 2d, and no more worthy or capable selection could have been made than is the appointment of Dr. Leonard Pearson to represent the Keystone State. Governor Hastings, as President of the State Live-stock Sanitary Board, directing, as he does, a work in our State that has never been undertaken by any other Commonwealth, bearing upon certain aspects of that all-important subject of tuberculosis, was quick to perceive the great good to be derived by having in touch with the world's most advanced students on this subject a delegate to present the work undertaken by our own State and to know of every line of investigation the world over. We are sure that no Governor of our State for many years has shown a keener appreciation of the welfare of all our people than the present Executive, and none who have grasped more quickly the intimate relations of our agricultural and livestock industries and their broad and far-reaching influences upon the welfare of our people physically, mentally, and morally, not to mention its gigantic importance to a large body of our people from a commercial point of view. *A wise decision. An excellent appointment.*

